# Skeletal Muscle‐Derived Extracellular Vesicles in Interorgan Communication During Aging

**DOI:** 10.1111/acel.70773

**Published:** 2026-10-15

**Authors:** Weiyi Jiang, Kenon Chua, Joe Young, Eng King Tan, Hong‐Wen Tang

**Affiliations:** ^1^ Program in Cancer and Stem Cell Biology Duke‐NUS Medical School Singapore Singapore; ^2^ Department of Orthopaedic Surgery Singapore General Hospital Singapore Singapore; ^3^ Institute of Molecular and Cell Biology (IMCB), Agency for Science, Technology and Research (A*STAR) Singapore Singapore; ^4^ Program in Neuroscience and Behavioural Disorders Duke‐NUS Medical School Singapore Singapore; ^5^ National Neuroscience Institute, Department of Neurology Singapore General Hospital Singapore Singapore

## Abstract

Skeletal muscle is increasingly recognized as a secretory organ that communicates with distant tissues through the release of bioactive factors, including extracellular vesicles (EVs). During aging, muscle‐derived EVs have emerged as important mediators of interorgan communication, influencing tissue homeostasis, regeneration, metabolism, and disease progression. Recent studies demonstrate that skeletal muscle EVs carry diverse cargos such as proteins, lipids, metabolites, and regulatory RNAs that can modulate the function of recipient cells in multiple organs. In particular, aging‐associated alterations in EV biogenesis, cargo composition, and secretion may contribute to chronic inflammation, stem cell dysfunction, metabolic decline, and age‐related pathologies. In this review, we summarize current advances in skeletal muscle‐derived EV biology and their roles in interorgan communication during aging. We discuss mechanisms regulating EV formation and cargo loading, their impact on stem cell function and tissue regeneration, and their involvement in aging‐related diseases. We also highlight the translational potential of muscle EVs as biomarkers and therapeutic targets. Together, these findings identify muscle‐derived EVs as key regulators of systemic aging and interorgan signaling.

## Graphical abstract

Skeletal muscle‐derived extracellular vesicles mediate interorgan communication with bone, brain, and tumors. Aging and exercise reshape EV release and cargo, shifting the balance between protective and maladaptive signals and highlighting muscle EV communication as a potential therapeutic target in age‐related disease.

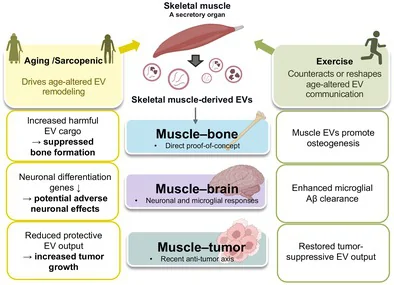

## 1 Introduction

Skeletal muscle is the largest organ in the human body and plays essential roles in locomotion, metabolism, and whole‐body homeostasis (Han et al. 2022; Gopal Krishnan et al. 2025). Beyond its traditional role as a contractile tissue, skeletal muscle is now recognized as a secretory organ that contributes to interorgan communication (Iglesias 2025; Severinsen and Pedersen 2020). Muscle homeostasis depends on the coordinated regulation of muscle fibers, stem cells, metabolism, and systemic signaling pathways (Goh et al. 2024; Lin, Rose, et al. 2026). During aging, this balance progressively declines, leading to sarcopenia, an age‐associated condition characterized by the loss of muscle mass and strength (Goh et al. 2024; Choy et al. 2025; Gou et al. 2026). Sarcopenia not only impairs mobility and quality of life but is also increasingly linked to dysfunction in distal tissues and age‐related diseases (Liu et al. 2025). Muscle aging also involves changes in regenerative capacity and tissue remodeling, both of which depend in part on resident muscle stem cells, known as satellite cells. Fry et al. showed that satellite cell depletion in sedentary male mice impaired regeneration and increased muscle fibrosis during aging without accelerating sarcopenia (Fry et al. 2015).

Early models of muscle‐organ communication focused mainly on soluble myokines, which are muscle‐derived factors capable of acting on distant tissues (Severinsen and Pedersen 2020). The discovery that skeletal muscle also releases extracellular vesicles expands this framework beyond soluble factor signaling (Fernández‐Rhodes et al. 2024; Jia et al. 2024; Rome et al. 2019). Extracellular vesicles are membrane‐delimited particles whose molecular cargo can reflect the state of their cell of origin and alter the phenotype of recipient cells (Welsh et al. 2024). In skeletal muscle, these vesicles can carry proteins, lipids, nucleic acids, and metabolites, allowing muscle to export complex molecular cargo rather than isolated soluble mediators (Fernández‐Rhodes et al. 2024; Jia et al. 2024). Because exercise, disease, and aging can alter muscle activity and muscle homeostasis, changes in skeletal muscle EV release or cargo may provide a route through which muscle state is communicated to distal tissues (Jia et al. 2024; Vechetti Jr., Valentino, et al. 2021).

This review examines skeletal muscle‐derived EVs as mediators of communication between muscle and distal tissues during aging. We first outline evidence for muscle EV release into the circulation and local signaling within skeletal muscle, before examining muscle–bone, muscle–brain, and muscle–tumor communication and EV effects on macrophages. We then consider how aging alters this communication system by modifying muscle EV release, cargo composition, and functional effects on recipient tissues. Where evidence is derived from mixed circulating EV populations rather than defined muscle‐derived EVs, we distinguish these studies from direct muscle EV evidence (Rome et al. 2019; Welsh et al. 2024; Vechetti Jr., Valentino, et al. 2021).

## 2 Muscle‐Derived EVs in Interorgan Communication

Skeletal muscle‐derived EVs can enter the circulation, providing a route for communication with distal tissues. Estrada et al. used a myofiber‐specific fluorescent reporter mouse to demonstrate that EVs released by skeletal muscle fibers reach the blood (Estrada et al. 2022). Complementary work by Vechetti et al. linked mechanical loading of skeletal muscle to EV‐associated miR‐1 delivery to white adipose tissue and increased lipolysis, using circulating EV preparations together with myotube‐derived EV experiments (Vechetti Jr., Peck, et al. 2021). These studies support the capacity of muscle EVs to participate in interorgan communication.

In addition to myofibers, resident satellite cells and their myogenic progeny release EVs that influence neighboring cells within skeletal muscle. Fry et al. showed that myogenic progenitor cell‐derived EVs deliver miR‐206 to fibrogenic cells, repressing Rrbp1 and limiting excessive extracellular matrix deposition during overload‐induced muscle growth (Fry et al. 2017). Extending this evidence to myofiber recipients, Murach et al. combined in vivo EV tracking with a model of delayed satellite cell fusion to provide evidence for satellite‐cell EV transfer to muscle fibers during mechanical overload (Murach et al. 2020). These findings identify satellite cells and their progeny as contributors to local EV‐mediated muscle remodeling. Their potential contribution to EV‐mediated responses in distal tissues during aging warrants further investigation.

### 2.1 Muscle–Bone Communication: A Proof‐Of‐Concept for EV‐Mediated Distal Tissue Regulation

Beyond these local interactions within skeletal muscle, muscle–bone communication provides a direct proof‐of‐concept for EV‐mediated distal tissue regulation. In this axis, recipient bone‐lineage cells and functional outcomes are clearly defined, allowing EV cargo transfer to be linked to osteogenic differentiation, bone‐forming activity, and bone loss (Ma et al. 2023; Shao et al. 2026; Xu et al. 2018). This evidence base therefore provides a useful starting point for establishing how skeletal muscle‐derived EVs can reach distal tissues and transmit state‐dependent signals beyond soluble myokine signaling.

Early in vitro evidence showed that EVs released from C2C12 myoblasts promote osteogenic differentiation of MC3T3‐E1 pre‐osteoblasts by transferring miR‐27a‐3p, which suppresses APC and activates β‐catenin signaling in recipient cells (Xu et al. 2018). This finding demonstrated in a defined culture system that muscle cell‐derived EV cargo can modify osteogenic programs in bone‐lineage cells, supporting EV cargo transfer as a potential mechanism of muscle–bone communication beyond soluble myokine signaling (Xu et al. 2018).

In vivo evidence further supports skeletal muscle‐derived EVs as functional mediators of muscle‐to‐bone communication. Ma et al. identified skeletal muscle‐derived EVs that enter the circulation, reach bone, and are taken up by bone marrow mesenchymal stromal cells (Ma et al. 2023). These EVs promoted osteogenic differentiation and protected mice from disuse osteoporosis, in part by delivering glycolytic enzymes such as lactate dehydrogenase A to enhance glycolytic metabolism in recipient stromal cells (Ma et al. 2023). Together, these findings show that muscle EVs can transmit metabolic cargo to bone‐lineage cells and reshape their differentiation state.

Muscle–bone EV signaling can also become deleterious when skeletal muscle is pathologically remodeled. Shao et al. showed that EVs derived from atrophic skeletal muscle are taken up by osteoblasts and suppress bone‐forming activity; conversely, muscle‐targeted blockade of EV generation restores osteoblast activity and alleviates bone loss (Shao et al. 2026), indicating that the muscle‐to‐bone EV route can transmit either supportive or inhibitory signals depending on muscle state.

Together, these studies establish muscle–bone communication as a direct proof‐of‐concept for EV‐mediated distal tissue regulation. Skeletal muscle‐derived EVs can enter the circulation, reach bone‐lineage recipient cells, and transfer osteogenic or inhibitory cargo, supporting muscle–bone EV signaling as a state‐dependent interorgan communication route.

### 2.2 Muscle–Brain Communication: EV‐Mediated Regulation of Neuronal and Brain Homeostatic States

#### 2.2.1 Muscle‐Derived EVs and Neuronal Plasticity

Beyond musculoskeletal tissues, neuronal recipient models provide evidence that muscle‐derived EVs can influence neuronal survival, morphology, and activity. Madison et al. showed that EVs released from C2C12 muscle cells enhanced survival and neurite outgrowth of NSC‐34 motor neuron‐like cells, indicating that muscle cell‐derived vesicles can modify neuronal behavior in a defined culture system (Madison et al. 2014). Although this model does not establish long‐range muscle‐to‐brain communication in vivo, it identifies neuronal cells as EV‐responsive recipients of muscle cell‐derived vesicles and supports the plausibility of muscle‐to‐neuron EV signaling (Madison et al. 2014).

Huang K.‐Y. et al. later used an in vitro engineered neuromuscular tissue model, in which myoblast‐derived skeletal muscle sheets were innervated by motor neurons differentiated from neural stem cells, to examine how neuromuscular activity conditions muscle‐derived secretions (Huang, Upadhyay, et al. 2024). In this model, innervation and glutamate stimulation increased EV release from skeletal muscle and altered exosomal miRNA cargo, with desmin‐based analysis supporting skeletal muscle as the major source of EVs in the conditioned medium (Huang, Upadhyay, et al. 2024). Functionally, conditioned medium from innervated, glutamate‐stimulated muscle increased the neuron‐to‐astrocyte ratio, dendritic branching, axonal transport, and spontaneous network activity in primary hippocampal cultures (Huang, Upadhyay, et al. 2024). Purified EVs from this medium also enhanced spontaneous neuronal network activity, supporting an EV contribution to this response (Huang, Upadhyay, et al. 2024).

A second study further linked skeletal muscle‐derived exosomal cargo to neuronal protection under injury conditions. Huang M. et al. reported that preconditioning exercise increased exosomal miR‐484, which was mainly derived from skeletal muscle, and that this exosomal miRNA reduced neuronal ferroptosis after ischemic brain injury by suppressing the ACSL4 pathway (Huang, Cheng, et al. 2024). Exosomes derived from L6 rat skeletal muscle myoblasts localized to the brain and were taken up by neurons in the ischemic penumbra (Huang, Cheng, et al. 2024). In the same study, inhibition of skeletal muscle miR‐484 production weakened the neuroprotective effect of preconditioning exercise (Huang, Cheng, et al. 2024). Together, these studies support the concept that skeletal muscle‐derived EVs can regulate neuronal survival, structural plasticity, and activity‐related phenotypes, providing experimental support for EV‐mediated muscle–brain communication.

#### 2.2.2 Brain Processes Relevant to Muscle EV‐Mediated Communication

In addition to neurons, microglia can respond to skeletal muscle‐derived EVs in the brain. In an Alzheimer's disease mouse model, Lin et al. showed that exercise‐induced muscle EVs were taken up by microglia and promoted a disease‐associated microglial state associated with enhanced amyloid‐β plaque clearance (Lin, Shao, et al. 2026). These neuronal and microglial studies provide direct evidence of muscle EV effects on brain‐related recipient cells, whereas broader muscle–brain studies define additional processes relevant to future EV‐focused work. Studies of muscle–brain crosstalk show that physical activity and muscle‐derived systemic factors regulate hippocampal function, learning and memory, mood‐related behaviors, and neurogenesis‐related pathways (Isaac et al. 2021; Pedersen 2019). Exercise‐related exerkines have also been linked to synaptic potentiation and neuroplasticity programs (Vints et al. 2022). In addition, genetic enhancement of skeletal muscle lysosomal and mitochondrial function through TFEB reduced CNS neuroinflammation, tau‐associated pathological features, and cognitive decline in aging and neurodegenerative disease models (Matthews et al. 2023).

These broader findings identify relevant brain outcomes, but do not by themselves establish that muscle‐derived EVs mediate them. Therefore, they should be used to define brain outcomes that may be tested in future EV‐focused studies, rather than as direct evidence that muscle‐derived EVs mediate muscle–brain communication. Relevant recipient readouts include neuronal plasticity, neuroimmune regulation, and neurodegenerative pathology. Within this framework, muscle‐derived EVs represent one possible cargo‐delivery route, alongside soluble myokines and other exerkines, through which skeletal muscle state may influence neural and glial environments.

### 2.3 Muscle‐Derived EVs in Tumor‐Related Communication

Beyond bone and brain‐related recipient cells, tumor cells represent another potential target of skeletal muscle‐derived EV signaling. In a recent proof‐of‐principle in vitro study, Mannaperuma et al. showed that EVs isolated from differentiated C2C12 myocytes were taken up by CMT 64/61 murine lung carcinoma cells and reduced carcinoma‐cell viability, proliferation‐associated readouts, and migration, while increasing cytotoxicity and apoptotic markers including caspase‐3 and caspase‐9 activity (Mannaperuma et al. 2025). These effects were accompanied by reduced lysosomal signal and impaired mitochondrial membrane potential; proteomic analysis further identified decorin as an enriched myocyte EV cargo and a candidate contributor to the anti‐tumor effect (Mannaperuma et al. 2025).

More direct in vivo evidence was provided by Goh et al., who showed that skeletal muscle‐derived EVs suppress tumorigenesis in *Drosophila* and mouse cancer models (Goh, Lee, Gou, et al. 2026). This study supports muscle as an anti‐tumor secretory organ and shows that tumor‐suppressive activity is at least partly mediated by muscle‐derived EV cargo, including miR‐7a‐5p‐mediated inhibition of TEAD1 signaling in tumor cells (Goh, Lee, Gou, et al. 2026). These findings extend the muscle–tumor EV axis from a defined myocyte–carcinoma cell system to organismal cancer models.

This experimental evidence is consistent with broader exercise‐oncology frameworks proposing that contracting skeletal muscle‐released EVs may contribute to systemic anti‐cancer effects (Pinto et al. 2024; Vitucci et al. 2023). Muscle‐derived EVs have also been proposed to influence tumor microenvironment remodeling and pre‐metastatic niche formation, while direct evidence linking muscle EVs to cancer metastasis remains scarce (Darkwah et al. 2021). Thus, current evidence most strongly supports an anti‐tumor muscle EV axis, while broader roles in tumor microenvironment remodeling and metastasis remain less directly established.

### 2.4 Muscle‐Derived EVs and Macrophage Responses

In addition to the tissue‐specific effects described above, muscle cell‐derived EVs can modulate inflammatory responses in cultured macrophages. Macrophages contribute to both skeletal muscle atrophy and regeneration, depending on their inflammatory state and the tissue context (Liu, Shen, et al. 2026; Shang et al. 2026; Chan et al. 2026). In a culture study, Yamaguchi et al. found that EVs from C2C12 myotubes reduced LPS‐induced inflammatory gene expression in bone marrow‐derived macrophages, alongside increased IRG1 expression and itaconate production (Yamaguchi et al. 2023). This study supports an immunoregulatory effect of myotube EVs in vitro; whether similar effects occur in macrophages of distal tissues or change with aging remains to be established.

Skeletal muscle‐derived EVs have been implicated in muscle–bone, muscle–brain, and muscle–tumor communication. Muscle–bone communication currently provides the clearest proof‐of‐concept for distal EV‐mediated tissue regulation, with defined EV cargo linked to osteogenic outcomes. Muscle–brain evidence includes neuronal responses in culture and injury models, as well as microglial responses in an Alzheimer's disease mouse model. Muscle–tumor studies provide recent evidence for anti‐tumor EV signaling through miR‐7a‐5p‐mediated signaling and candidate cargo such as decorin.

## 3 Aging as a Modifier of Muscle EV‐Mediated Communication

The organ contexts discussed above indicate that skeletal muscle‐derived EVs can act on multiple recipient cell types, including bone‐lineage cells, neuronal cells, tumor cells, and cultured macrophages with different levels of evidence across these axes (Figure 1). The next question is how aging changes this muscle‐derived vesicular communication system at the level of EV production, cargo composition, and functional output.

**FIGURE 1:**
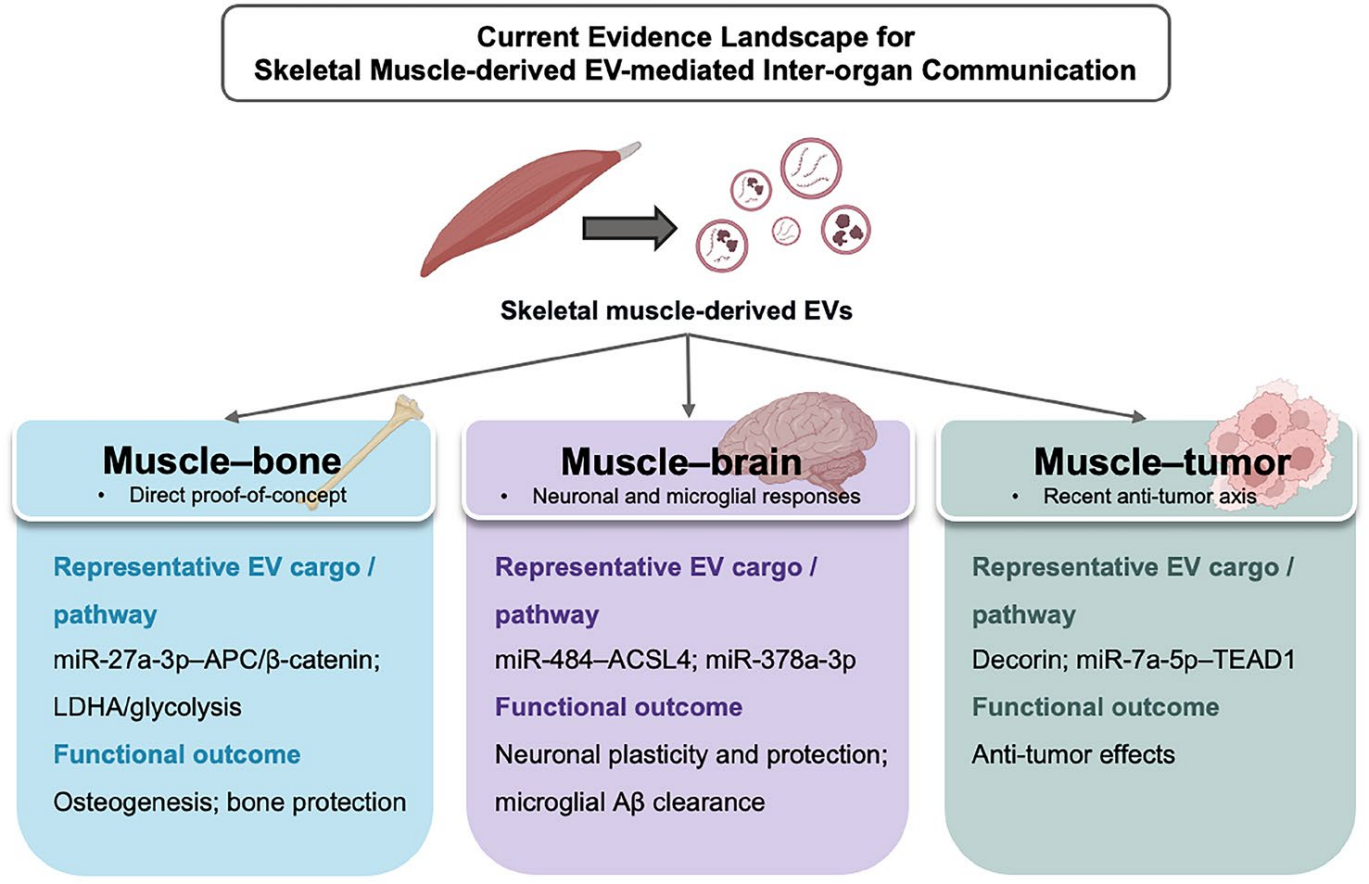
Current evidence landscape for skeletal muscle‐derived EV‐mediated interorgan communication.

### 3.1 Aging‐Associated Remodeling of Muscle EV Communication Across Distal Tissues

Aging remodels skeletal muscle‐derived EV communication by changing both vesicle cargo and downstream recipient‐cell responses. In human primary skeletal muscle cells, EVs from older donors showed altered biochemical and proteomic signatures, including increased protein, lipid, and hypoxanthine signals and enrichment of proteins associated with oxidative stress and DNA/RNA mutagenesis, even when basic EV parameters showed limited changes (Fernández‐Rhodes et al. 2024; Jiang, Chua, et al. 2026). These findings indicate that aging may change the molecular message carried by muscle EVs even when obvious changes in vesicle abundance or size are not detected.

This age‐related EV remodeling has been most clearly demonstrated in muscle–bone communication. Fulzele et al. showed that α‐sarcoglycan‐positive circulating EVs, enriched as a muscle‐associated population, contain increased miR‐34a with age, and that miR‐34a‐enriched EVs from stressed muscle cells reduce bone marrow stromal cell viability and promote senescence (Fulzele et al. 2019). Shao et al. further demonstrated that aged/atrophic skeletal muscle‐derived EVs are taken up by osteoblasts and suppress bone formation through miR‐125a‐5p‐mediated inhibition of the Sirt7–Sp7 osteogenic axis (Shao et al. 2026). Together, these studies indicate that aging or atrophy can shift muscle EV cargo toward senescence‐ or atrophy‐associated signals that impair bone‐lineage cell function.

A similar principle may extend to muscle–brain communication, although the evidence is less complete. Aging‐ and atrophy‐associated miR‐29b‐3p has been reported to increase in muscle and plasma. C2C12‐derived EVs carrying this miRNA were taken up by SH‐SY5Y cells and reduced the expression of neuronal differentiation‐related genes. In separate experiments, miR‐29b‐3p overexpression inhibited neuronal differentiation (Yang et al. 2020). This finding suggests that age‐ or atrophy‐associated muscle EV miRNA cargo can influence neuronal phenotypes in cell‐based models, raising the possibility that aged muscle remodels muscle–brain communication through altered EV cargo, although direct in vivo transfer to brain cells remains to be established.

Recent evidence indicates that aging‐associated sarcopenic remodeling can also alter EV‐mediated communication in the muscle–tumor axis. Goh et al. showed that healthy skeletal muscle releases EVs with tumor‐suppressive activity, whereas sarcopenic muscle exhibits reduced EV secretion and altered EV cargo, leading to a loss of this protective effect (Goh, Lee, Gou, et al. 2026). This loss of protective EV function was linked to reduced EV‐enriched miR‐7a‐5p in aged muscle, which weakens miR‐7a‐5p‐mediated inhibition of TEAD1 signaling in tumor cells (Goh, Lee, Gou, et al. 2026). Mechanistically, the authors identified a NOTCH–SDC2 pathway that regulates muscle EV biogenesis and declines with age, connecting sarcopenic muscle remodeling to impaired anti‐tumor EV output (Goh, Lee, Gou, et al. 2026).

Beyond the changes in EV release and miRNA cargo described above, mitochondrial DNA (mtDNA) may provide another potential link between muscle‐derived vesicles and responses in distal tissues. One pathway through which this cargo could act is cGAS–STING signaling. In a liver‐to‐muscle model, EVs carrying mtDNA activated cGAS–STING in skeletal muscle macrophages and promoted inflammatory muscle injury (Fan et al. 2025). In skeletal muscle, sustained cGAS–STING activation has been linked to inflammation and atrophy, whereas moderate activation during exercise may support metabolic adaptation (Jiang, Ji, et al. 2026). This pathway also regulates macrophage activation and polarization (Liu, Zhu, et al. 2026), and macrophages contribute to both muscle regeneration and atrophy depending on their state (Liu, Shen, et al. 2026; Shang et al. 2026). More directly relevant to muscle‐to‐brain communication, mtDNA‐rich mitochondrial‐derived vesicles released from skeletal muscle after excessive vigorous exercise were shown to activate cGAS–STING in hippocampal neurons (Huang et al. 2026). Together, these findings suggest that EV‐associated mtDNA can engage this pathway in recipient cells. Whether aging changes mtDNA cargo in muscle‐derived EVs and thereby alters cGAS–STING signaling in distal tissues remains unknown.

These potential effects of EV cargo in recipient cells also raise the question of how aging might change cargo in the muscle of origin. ER stress and the unfolded protein response (UPR), which have been implicated in skeletal muscle atrophy, may provide one possible route (Ji et al. 2025). In a lipotoxicity model, McNally et al. showed that stressed myotubes released EVs containing long‐chain ceramides that induced UPR signaling in recipient myotubes; the production of these lipids was linked to the PERK branch of the UPR (McNally et al. 2022). This study demonstrates that a stress‐related pathway in muscle can be linked to EV cargo and responses in other muscle cells. Future studies can test whether aging‐associated changes in muscle ER stress and UPR signaling alter EV release or cargo, whether these EVs affect distal tissues, and whether modulation of the relevant UPR branch changes these effects.

Together, the bone‐, brain‐, and tumor‐related examples discussed above indicate that aging can remodel muscle EV communication by altering EV release or abundance, cargo composition, and recipient‐tissue responses. The additional studies suggest two mechanisms for future investigation: mtDNA–cGAS–STING signaling in recipient cells and ER stress–UPR‐associated changes in EV cargo in donor muscle. Whether either mechanism contributes to aging‐related effects in distal tissues remains to be established.

### 3.2 Exercise‐Sensitive Muscle EV Signaling in Aging

Exercise is widely regarded as an effective approach to improving age‐related skeletal muscle dysfunction (Choy et al. 2025). Given that aging alters muscle EV release and cargo, an important question is how exercise affects muscle EV communication during aging. Current evidence suggests two ways in which exercise may affect this communication: it can induce protective skeletal muscle‐derived EV signals that act on distal tissues, and it can modify EV cargo profiles that are already altered by aging. This distinction separates newly induced exercise‐responsive EV outputs from training‐associated remodeling of preexisting aged EV profiles.

#### 3.2.1 Exercise‐Induced Muscle EV Signals in Aging‐Related Tissue Protection

Exercise‐induced skeletal muscle EVs provide one route through which muscle activity may protect aging‐sensitive tissues. In an Alzheimer's disease mouse model, Lin et al. showed that swimming exercise promoted the secretion of skeletal muscle‐derived EVs, which were taken up by microglia through pinocytosis (Lin, Shao, et al. 2026). These exercise‐induced EVs enhanced disease‐associated microglial polarization and promoted amyloid‐β plaque clearance through miR‐378a‐3p‐mediated regulation of lipid metabolism (Lin, Shao, et al. 2026). Administration of EVs derived from miR‐378a‐overexpressing myotubes also alleviated cognitive impairment, supporting the potential to reproduce a protective exercise‐associated muscle EV signal (Lin, Shao, et al. 2026). This study suggests that exercise‐induced skeletal muscle EVs can modify microglial lipid metabolism and plaque‐clearance activity, providing direct evidence for EV‐mediated muscle–brain communication in an aging‐related neurodegenerative context.

#### 3.2.2 Exercise‐Mediated Remodeling of Age‐Altered Muscle EV Cargo

Exercise may also act by modifying EV profiles that are already disrupted by aging. Xhuti et al. reported that older individuals had reduced circulating exosome‐like vesicle markers, including CD9 and CD81, lower levels of EV‐associated miRNAs linked to skeletal muscle health, and reduced endogenous and secreted myomiRs in aged human myotube models at baseline (Xhuti et al. 2023). After resistance exercise training, several of these EV‐associated miRNAs increased toward levels observed in young individuals (Xhuti et al. 2023). Because this study examined circulating exosome‐like vesicles together with skeletal muscle and myotube miRNA profiles, it does not by itself establish direct muscle‐derived EV transfer in vivo. However, resistance training increased several circulating EV‐associated miRNAs, whereas skeletal muscle miRNA expression showed little training‐related change (Xhuti et al. 2023). These findings identify circulating EV‐associated miRNAs as candidate readouts of training responses, with their muscle origin and effects on distal tissues as priorities for future study. Complementing these circulating EV observations, Goh et al. showed that exercise increased muscle NOTCH–SDC2 signaling, EV release, and EV‐associated miR‐7a‐5p in aged mice, alongside reduced tumor growth (Goh, Lee, Gou, et al. 2026). Inhibiting muscle NOTCH signaling or reducing SDC2 weakened these responses, linking exercise to restoration of an age‐impaired muscle EV pathway (Goh, Lee, Gou, et al. 2026).

The studies above describe exercise‐induced protective muscle EV signals and training‐associated changes in age‐altered EV profiles. However, which metabolic pathways in skeletal muscle contribute to exercise‐related changes in EV cargo and function remains unclear. One candidate is the AMPK/SIRT1/PGC‐1α signaling network, which regulates muscle energy metabolism and contributes to exercise adaptation (Chen et al. 2025; Wang et al. 2026; Yan et al. 2022). Among these regulators, PGC‐1α has been directly examined in relation to muscle‐derived EVs. Kargl et al. showed that PGC‐1α overexpression in human myotubes did not increase EV release but increased the levels of antioxidant‐related mRNAs in their EVs. These EVs also enhanced angiogenesis‐related responses and protection against oxidative stress in cultured endothelial cells (Kargl et al. 2023). These findings suggest that the metabolic state of donor muscle cells can influence EV cargo and recipient‐cell responses. However, whether AMPK or SIRT1 helps regulate exercise‐related changes in muscle EV cargo and whether these EVs alter AMPK/SIRT1/PGC‐1α signaling in distal recipient cells, particularly during aging, remains to be established.

Together, these findings support a model in which aging and exercise regulate skeletal muscle‐derived EV communication by reshaping EV release, cargo composition, and EV biogenesis or molecular features, thereby modifying muscle‐to‐tissue signaling during aging. The PGC‐1α study further suggests that the metabolic state of donor muscle cells may influence EV cargo and function, although whether this mechanism contributes to exercise‐sensitive interorgan communication during aging remains to be established (Figure 2).

**FIGURE 2:**
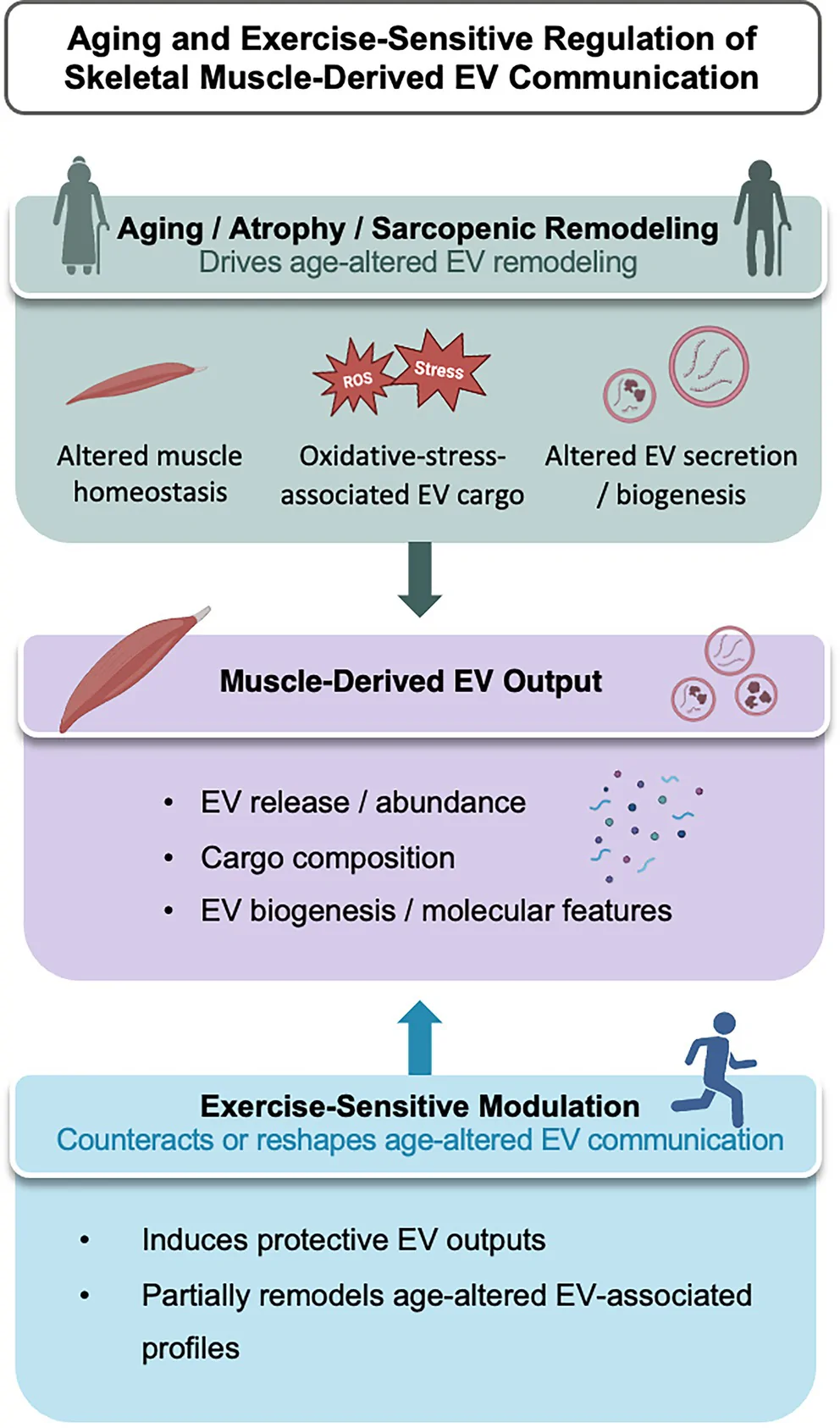
Aging and exercise‐sensitive regulation of skeletal muscle‐derived EV communication. Aging, atrophy, and sarcopenic remodeling alter skeletal muscle‐derived EV output by changing EV release, cargo composition, and EV biogenesis or molecular features, thereby modifying EV‐mediated interorgan communication with distal recipient tissues. Exercise may counteract this remodeling by inducing protective EV signals and partially restoring age‐altered EV‐associated profiles. This model summarizes how muscle state regulates EV‐mediated communication during aging.

## 4 Therapeutic Perspectives: Modulating Muscle EV Communication in Aging

The evidence discussed above supports a therapeutic framework that distinguishes protective from maladaptive muscle EV signals. Protective muscle EV signals that decline with aging or disease could be restored or mimicked, whereas maladaptive EV signals generated by aged or atrophic muscle could be selectively reduced. The feasibility of these approaches depends on reliable EV isolation and characterization, confirmation of muscle origin and causal cargo, and identification of the recipient cells affected. We first discuss strategies to restore protective signals, reduce maladaptive signals, and modulate muscle EV communication through exercise. We then consider how EV isolation and characterization affect the evidence for these strategies and their therapeutic development.

### 4.1 Restoring Protective Muscle EV Signals

One therapeutic direction is to restore beneficial muscle EV outputs that support distal tissue function. In bone, skeletal muscle‐derived EVs carrying glycolytic enzymes promote glycolytic metabolism and osteogenic differentiation in bone marrow stromal cells (Ma et al. 2023). In the brain, exercise‐induced skeletal muscle EVs carrying miR‐378a‐3p enhance microglial amyloid‐β plaque clearance and improve cognitive outcomes in an Alzheimer's disease model (Lin, Shao, et al. 2026). In tumor‐related contexts, healthy muscle EV cargo such as miR‐7a‐5p suppresses TEAD1 signaling in tumor cells, whereas aging‐associated sarcopenic remodeling reduces this protective EV output (Goh, Lee, Gou, et al. 2026). These examples identify candidate cargo–pathway pairs that could guide EV‐mimetic or cargo‐focused intervention strategies.

At present, direct evidence for engineering muscle‐derived EVs to reproduce these protective interorgan signals remains limited. The clearest example comes from the muscle–brain axis, where Lin et al. generated EVs from miR‐378a‐overexpressing C2C12 myotubes and administered these cargo‐enriched vesicles to Alzheimer's disease mice (Lin, Shao, et al. 2026). This strategy was designed to mimic an exercise‐induced protective skeletal muscle‐derived EV signal, and the treated mice showed increased brain miR‐378a‐3p levels, enhanced microglial plaque‐clearance activity, and improved cognitive performance (Lin, Shao, et al. 2026). This study therefore supports the feasibility of a muscle‐EV‐mimetic approach in which a defined protective miRNA cargo is enriched in myotube‐derived EVs and delivered in vivo.

More general muscle‐related EV engineering studies, such as EV‐mediated CRISPR/Cas9 delivery targeting miR‐29b in muscle atrophy models, show that EV cargo design can be used therapeutically in skeletal muscle disease contexts, but they do not directly model muscle‐derived EV communication with distal recipient tissues (Chen et al. 2022). Therefore, restoring protective muscle EV signals should currently be framed as an emerging therapeutic concept. Future strategies will need to use defined donor muscle cells or delivery systems, enrich causal cargo efficiently, verify delivery to the intended recipient cells, and limit the transfer of harmful cargo.

### 4.2 Blocking Maladaptive Aged‐Muscle EV Signals

A complementary strategy is to reduce EV signals generated by aged or atrophic muscle when they impair recipient tissues. This approach is best supported in bone‐related studies. miR‐34a increases with age in α‐sarcoglycan‐positive circulating EVs, and miR‐34a‐enriched muscle‐cell EVs promote senescence in bone marrow stromal cells (Fulzele et al. 2019). Aged/atrophic muscle EV miR‐125a‐5p also suppresses osteogenic differentiation through the Sirt7–Sp7 axis (Ismaeel et al. 2023). These findings suggest that pathogenic EV cargo, rather than EV release, may be a more selective therapeutic target.

Preclinical evidence supports intervention in this pathway: Shao et al. reduced age‐related bone loss by suppressing muscle EV generation or inhibiting miR‐125a‐5p in skeletal muscle (Shao et al. 2026). Future studies should test whether cargo‐directed interventions preserve beneficial muscle EV signals while reducing osteoblast inhibition. Broad inhibition of EV production would be difficult to apply therapeutically because skeletal muscle EVs can also carry protective cargo that promotes osteogenesis, supports brain function, or suppresses tumor growth. Future approaches will therefore need to distinguish harmful age‐associated cargo from beneficial vesicular communication. Possible intervention logic includes selectively reducing pathogenic cargo loading, blocking uptake by vulnerable recipient cells, or counterbalancing maladaptive EV signals with protective muscle‐EV‐mimetic inputs. An alternative strategy may be to target pathological remodeling of the donor muscle itself, thereby modifying EV output upstream. For example, celecoxib attenuated muscle atrophy and inflammation in models of denervation and diabetic sarcopenia (Deng et al. 2024; Zhang et al. 2022), while AAV‐mediated modulation of *Deaf1* improved age‐related sarcopenia (Lee et al. 2025). Whether these effects also change muscle EV release or cargo, and consequently alter signals reaching distal tissues, remains to be tested.

### 4.3 Exercise‐Informed Modulation of Muscle EV Communication

Exercise represents a physiological strategy for modulating muscle EV communication during aging. Unlike engineered EV approaches, exercise does not deliver a single predefined cargo. Instead, it may reshape the muscle secretory state by inducing protective EV outputs and by remodeling EV‐associated cargo profiles that are altered with age. The exercise‐related studies discussed above therefore have therapeutic relevance not only because exercise may directly modify muscle EV signaling but also because they identify EV‐associated readouts that can be used to evaluate or design future interventions.

From a therapeutic perspective, exercise‐responsive EV cargo can serve two roles. First, it may provide measurable biomarkers for evaluating whether an intervention restores a more youthful or protective muscle secretory state. Second, it may help identify cargo candidates for future muscle‐EV‐mimetic strategies. However, exercise‐associated circulating EV studies often involve mixed EV populations and do not always establish skeletal muscle as the direct vesicle source. Therefore, future work should combine exercise interventions with muscle‐origin tracing, cargo perturbation, and recipient‐tissue readouts to determine which exercise‐responsive EV signals are causal mediators of interorgan benefit.

Beyond exercise itself, mechanosensitive signaling may offer a way to modify muscle EV output. Piezo1 is a mechanosensitive cation channel that converts mechanical stimuli into intracellular signals in skeletal muscle (Xue et al. 2026). Reduced Piezo1 activity contributes to disuse‐induced muscle atrophy through the KLF15–IL‐6 pathway (Hirata et al. 2022). More directly, Bernareggi et al. showed that treatment of differentiated C2C12 myotubes with the Piezo1 agonist Yoda1 increased small EV release and altered EV protein cargo. EVs collected after treatment promoted myotube formation in culture (Annalisa et al. 2026). These findings indicate that pharmacological Piezo1 activation can modify EV output from cultured myotubes and influence local myogenic responses. Whether exercise produces similar Piezo1‐dependent EV changes, or whether these vesicles protect distal tissues during aging, remains to be established.

### 4.4 EV Isolation and Characterization: Implications for Interpreting Evidence and Developing Therapies

Each of these therapeutic directions depends on establishing that the vesicles come from skeletal muscle and that their cargo produces the observed effects in recipient cells. Therefore, the first methodological question is whether an effect attributed to muscle EVs is carried by the vesicles themselves. Different isolation methods recover different mixtures of EVs and non‐vesicular material. For example, differential ultracentrifugation removes cells and some larger particles before enriching EVs, but non‐vesicular particles with similar sedimentation properties can remain in the final preparation. Size‐exclusion chromatography separates many soluble proteins but may retain particles of similar size, including lipoproteins. Density gradients and field‐flow fractionation offer further ways to resolve particle populations (Welsh et al. 2024). Characterization should combine measurements of particle size and morphology with EV‐associated markers and assessment of relevant co‐isolates, such as plasma lipoproteins (Welsh et al. 2024). In a direct comparison using rat plasma, Takov et al. found that ultracentrifugation yielded fewer EVs but higher purity than size‐exclusion chromatography. Despite these differences, preparations containing equal numbers of total particles promoted similar levels of endothelial cell migration, although the size‐exclusion preparation had much less CD81, an EV marker (Takov et al. 2019). This result leaves open whether EVs, co‐isolated material, or both contributed to the response. Future muscle EV studies should therefore compare EV‐enriched and EV‐depleted fractions from the same starting material and test whether altering candidate EV cargo changes recipient‐cell responses. Such tests would strengthen the evidence for EV‐mediated effects and help identify procedures that preserve reproducible activity. For larger‐scale production, tangential flow filtration combined with size‐exclusion chromatography has yielded functional EV preparations from mesenchymal stromal cells (Li et al. 2025; Zhou et al. 2026). Applying such workflows to muscle cells will require testing whether scale‐up preserves the cargo and recipient‐cell activity of smaller research preparations.

Besides testing whether the observed effect is mediated by EVs, future studies also need to establish the vesicles' cell sources. For example, Goh et al. directly established the donor‐cell sources by collecting EVs from cultured C2C12 myotubes and isolated mouse myofibers (Goh, Lee, Gou, et al. 2026). A related protocol describes EV isolation and characterization from differentiated C2C12 cells (Goh, Lee, and Tang 2026). However, EVs collected from whole muscle tissue may also originate from resident non‐muscle cells, and tissue processing can affect the particles recovered (Crescitelli et al. 2025).

In blood, EVs from many tissues circulate together. Fulzele et al. therefore used α‐sarcoglycan‐based capture to enrich a muscle‐associated subset from serum and detected an age‐related increase in miR‐34a in this subset (Fulzele et al. 2019). This type of enrichment may help identify vesicle populations for biomarker studies or future selective interventions, but the marker and cargo would need further validation. The need for marker validation is illustrated by Ismaeel et al., who did not detect α‐sarcoglycan in the rat serum EV preparations they examined or miR‐1 in rat and human serum EVs under basal conditions. They also found that common EV markers were expressed by multiple cell types within skeletal muscle, highlighting the limits of using individual markers to establish muscle origin (Ismaeel et al. 2023). For small blood samples, microfluidic approaches may facilitate EV analysis, but identifying muscle‐derived EVs still depends on validated source markers (Welsh et al. 2024).

To trace muscle EVs directly, Estrada et al. used a myofiber‐specific fluorescent reporter mouse and detected myofiber‐derived EVs in the circulation (Estrada et al. 2022). In a separate study tracing EVs to a distal tissue, Shao et al. used muscle‐specific reporter tracing to examine the transfer of muscle‐fiber EVs to bone cells in vivo (Shao et al. 2026). Together, these studies provide evidence for two distinct steps: release from muscle fibers into the blood and delivery to recipient cells. For therapeutic development, future studies should use defined donor muscle cells, test whether EV batches retain consistent cargo and biological activity, and verify delivery to the intended recipient cells after administration. These studies could help identify reproducible preparations and determine whether administered EVs reach the targets suggested by endogenous muscle EV tracing.

## 5 Conclusions

Skeletal muscle‐derived EVs provide a vesicular route through which muscle can communicate with distal tissues. Evidence from muscle–bone, muscle–brain, and muscle–tumor studies indicate that these vesicles can transfer functional cargo to recipient cells and influence tissue‐specific programs, including osteogenic differentiation, neuronal activity or protection, microglial amyloid‐β plaque clearance, and tumor growth. In culture, muscle cell‐derived EVs also alter macrophage inflammatory responses, although whether this occurs in distal tissues during aging remains to be established. Aging modifies this communication route by altering muscle EV release, cargo composition, and downstream effects on recipient tissues. In this context, aged or atrophic muscle may generate maladaptive EV signals that impair distal recipient tissues, whereas exercise or healthy muscle states may preserve protective muscle‐to‐tissue communication.

These findings support a therapeutic framework in which muscle EV communication could be modulated in two complementary ways: restoring protective vesicular signals that decline with aging or disease, and reducing maladaptive signals produced by aged or atrophic muscle. Engineered or cargo‐enriched muscle‐derived EVs, as well as muscle‐EV‐mimetic systems, represent one possible future strategy for this purpose. These approaches could be designed to reproduce selected protective muscle EV signals, such as defined miRNA or protein/enzyme cargo, while limiting the transfer of harmful aged‐muscle EV components.

However, most muscle EV axes still require stronger evidence that the vesicles originate from skeletal muscle, reach the proposed recipient cells, and account for the observed effects. Future studies should compare EV‐enriched and EV‐depleted fractions, test candidate cargo, and trace muscle EVs to recipient tissues to determine how these effects change with aging. For engineered EV or EV‐mimetic interventions, additional requirements include efficient cargo enrichment, preservation or reconstruction of tissue‐targeting properties, dosing and safety evaluation, and direct comparison with naturally produced muscle EVs. These criteria will be essential for evaluating muscle‐derived EVs not only as biomarkers of aging‐related muscle–tissue communication, but also as rational intervention targets for aging‐related interorgan dysfunction.

## Author Contributions

Weiyi Jiang and Kenon Chua led the literature search and manuscript drafting. Joe Young contributed to literature collection, selected drafting, and figure/table preparation. Eng King Tan assisted with literature organization and contributed to conceptual design. Hong‐Wen Tang supervised the review, provided overall guidance, and finalized the manuscript. All authors read and approved the final manuscript.

## Funding

This work was supported by Singapore's Ministry of Education AcRF Award (FY2025‐MOET1‐0004 to H.‐W.T.), Diana Koh Innovative Cancer Research Award (Duke‐NUS‐DKICRA/2024/0001, to H.‐W.T.), and National Medical Research Council (NMRC) (MOH‐001208‐00 and MOH‐001885‐00 to H.‐W.T.).

## Conflicts of Interest

The authors declare no conflicts of interest.

## Data Availability

Data sharing not applicable to this article as no datasets were generated or analysed during the current study.

## References

[acel70773-bib-0001] Annalisa, B. , Z. W. Ru , S. S. Laura , et al. 2026. “PIEZO1 Channels Modulate the Small Extracellular Vesicle Release in C2C12 Cells.” Journal of Cellular Physiology 241: e70155. 10.1002/jcp.70155 PMC12989915 41834672

[acel70773-bib-0002] Chan, M. , P. Low , S. C. Ong , et al. 2026. “Inflammaging and Osteoarthritis ‐ Therapeutic Opportunities.” Frontiers in Immunology 17: 1850495. 10.3389/fimmu.2026.1850495 PMC13337404 42416050

[acel70773-bib-0003] Chen, J. , B. Liu , X. Yao , et al. 2025. “AMPK/SIRT1/PGC‐1alpha Signaling Pathway: Molecular Mechanisms and Targeted Strategies From Energy Homeostasis Regulation to Disease Therapy.” CNS Neuroscience & Therapeutics 31: e70657. 10.1111/cns.70657 PMC12635776 41268687

[acel70773-bib-0004] Chen, R. , W. Yuan , Y. Zheng , et al. 2022. “Delivery of Engineered Extracellular Vesicles With miR‐29b Editing System for Muscle Atrophy Therapy.” Journal of Nanobiotechnology 20: 304. 10.1186/s12951-022-01508-4 PMC9235146 35761332

[acel70773-bib-0005] Choy, S. M. , K. Y. Goh , W. X. Lee , et al. 2025. “Exercise Suppresses DEAF1 to Normalize mTORC1 Activity and Reverse Muscle Aging.” Proceedings of the National Academy of Sciences of the United States of America 122: e2508893122. 10.1073/pnas.2508893122 PMC12685040 41284871

[acel70773-bib-0006] Crescitelli, R. , Y. Huang , A. Hendrix , et al. 2025. “Recommendations for Studying In Situ Extracellular Vesicles From Solid Tissue.” Journal of Extracellular Vesicles 14: e70185. 10.1002/jev2.70185 PMC12606037 41220186

[acel70773-bib-0007] Darkwah, S. , E. J. Park , P. K. Myint , et al. 2021. “Potential Roles of Muscle‐Derived Extracellular Vesicles in Remodeling Cellular Microenvironment: Proposed Implications of the Exercise‐Induced Myokine, Irisin.” Frontiers in Cell and Developmental Biology 9: 634853. 10.3389/fcell.2021.634853 PMC7892973 33614663

[acel70773-bib-0008] Deng, C. , C. Lu , K. Wang , et al. 2024. “Celecoxib Ameliorates Diabetic Sarcopenia by Inhibiting Inflammation, Stress Response, Mitochondrial Dysfunction, and Subsequent Activation of the Protein Degradation Systems.” Frontiers in Pharmacology 15: 1344276. 10.3389/fphar.2024.1344276 PMC10834620 38313305

[acel70773-bib-0009] Estrada, A. L. , Z. J. Valenti , G. Hehn , et al. 2022. “Extracellular Vesicle Secretion Is Tissue‐Dependent Ex Vivo and Skeletal Muscle Myofiber Extracellular Vesicles Reach the Circulation In Vivo.” American Journal of Physiology. Cell Physiology 322: C246–C259. 10.1152/ajpcell.00580.2020 PMC8816621 34910603

[acel70773-bib-0010] Fan, X. , Y. Peng , B. Li , et al. 2025. “Liver‐Secreted Extracellular Vesicles Promote Cirrhosis‐Associated Skeletal Muscle Injury Through mtDNA‐cGAS/STING Axis.” Advanced Science 12: e2410439. 10.1002/advs.202410439 PMC11884600 39804962

[acel70773-bib-0011] Fernández‐Rhodes, M. , E. Buchan , S. Gagnon , et al. 2024. “Extracellular Vesicles May Provide an Alternative Detoxification Pathway During Skeletal Muscle Myoblast Ageing.” Journal of Extracellular Biology 3: e171. 10.1002/jex2.171 PMC11336379 39169919

[acel70773-bib-0012] Fry, C. S. , T. J. Kirby , K. Kosmac , J. J. McCarthy , and C. A. Peterson . 2017. “Myogenic Progenitor Cells Control Extracellular Matrix Production by Fibroblasts During Skeletal Muscle Hypertrophy.” Cell Stem Cell 20: 56–69. 10.1016/j.stem.2016.09.010 PMC5218963 27840022

[acel70773-bib-0013] Fry, C. S. , J. D. Lee , J. Mula , et al. 2015. “Inducible Depletion of Satellite Cells in Adult, Sedentary Mice Impairs Muscle Regenerative Capacity Without Affecting Sarcopenia.” In Nat Med, vol. 21, 76–80. United States. 10.1038/nm.3710 PMC4289085 25501907

[acel70773-bib-0014] Fulzele, S. , B. Mendhe , A. Khayrullin , et al. 2019. “Muscle‐Derived miR‐34a Increases With Age in Circulating Extracellular Vesicles and Induces Senescence of Bone Marrow Stem Cells.” Aging 11: 1791–1803. 10.18632/aging.101874 PMC6461183 30910993

[acel70773-bib-0015] Goh, K. Y. , W. X. Lee , S. M. Choy , et al. 2024. “FOXO‐Regulated DEAF1 Controls Muscle Regeneration Through Autophagy.” Autophagy 20: 2632–2654. 10.1080/15548627.2024.2374693 PMC11587838 38963021

[acel70773-bib-0016] Goh, K. Y. , W. X. Lee , Q. Gou , et al. 2026. “Sarcopenia Promotes Tumorigenesis by Disrupting NOTCH‐SDC2‐Regulated Biogenesis of Muscle‐Derived Extracellular Vesicles.” Nature Communications 17: 5750. 10.1038/s41467-026-72410-y PMC13324703 42045191

[acel70773-bib-0017] Goh, K. Y. , W. X. Lee , and H. W. Tang . 2026. “Isolation and Biophysical Characterization of Extracellular Vesicles Released by Myocytes.” Bio‐Protocol 16: e5698. 10.21769/BioProtoc.5698 PMC13200068 42199476

[acel70773-bib-0018] Gopal Krishnan, P. D. , W. X. Lee , K. Y. Goh , et al. 2025. “Transcriptional Regulation of Autophagy in Skeletal Muscle Stem Cells.” Disease Models & Mechanisms 18: DMM052007. 10.1242/dmm.052007 PMC11849978 39925192

[acel70773-bib-0019] Gou, Q. , S. C. Ong , W. X. Lee , P. D. G. Krishnan , and H.‐W. Tang . 2026. “Fine‐Tuning Autophagy in Skeletal Muscle: From Homeostasis to Muscle Wasting Disorders.” Aging and Disease. 10.14336/ad.2026.0170. 41980220

[acel70773-bib-0020] Han, X. , K. Y. Goh , W. X. Lee , S. M. Choy , and H. W. Tang . 2022. “The Importance of mTORC1‐Autophagy Axis for Skeletal Muscle Diseases.” International Journal of Molecular Sciences 24: 297. 10.3390/ijms24010297 PMC9820406 36613741

[acel70773-bib-0021] Hirata, Y. , K. Nomura , D. Kato , et al. 2022. “A Piezo1/KLF15/IL‐6 Axis Mediates Immobilization‐Induced Muscle Atrophy.” Journal of Clinical Investigation 132: 1–13. 10.1172/JCI154611 PMC9159676 35290243

[acel70773-bib-0022] Huang, K. Y. , G. Upadhyay , Y. Ahn , et al. 2024. “Neuronal Innervation Regulates the Secretion of Neurotrophic Myokines and Exosomes From Skeletal Muscle.” Proceedings of the National Academy of Sciences of the United States of America 121: e2313590121. 10.1073/pnas.2313590121 PMC11087749 38683978

[acel70773-bib-0023] Huang, M. , S. Cheng , Z. Li , et al. 2024. “Preconditioning Exercise Inhibits Neuron Ferroptosis and Ameliorates Brain Ischemia Damage by Skeletal Muscle‐Derived Exosomes via Regulating miR‐484/ACSL4 Axis.” Antioxidants & Redox Signaling 41: 769–792. 10.1089/ars.2023.0492 38545792

[acel70773-bib-0024] Huang, Y. , B. Hu , Y. Liu , et al. 2026. “Excessive Vigorous Exercise Impairs Cognitive Function Through a Muscle‐Derived Mitochondrial Pretender.” Cell Metabolism 38: 281–297.e211. 10.1016/j.cmet.2025.11.002 41344322

[acel70773-bib-0025] Iglesias, P. 2025. “Muscle in Endocrinology: From Skeletal Muscle Hormone Regulation to Myokine Secretion and Its Implications in Endocrine‐Metabolic Diseases.” Journal of Clinical Medicine 14: 4490. 10.3390/jcm14134490 PMC12249830 40648864

[acel70773-bib-0026] Isaac, A. R. , R. A. S. Lima‐Filho , and M. V. Lourenco . 2021. “How Does the Skeletal Muscle Communicate With the Brain in Health and Disease?” Neuropharmacology 197: 108744. 10.1016/j.neuropharm.2021.108744 34363812

[acel70773-bib-0027] Ismaeel, A. , D. W. van Pelt , Z. R. Hettinger , et al. 2023. “Extracellular Vesicle Distribution and Localization in Skeletal Muscle at Rest and Following Disuse Atrophy.” Skeletal Muscle 13: 6. 10.1186/s13395-023-00315-1 PMC9999658 36895061

[acel70773-bib-0028] Ji, Y. , Q. Jiang , B. Chen , et al. 2025. “Endoplasmic Reticulum Stress and Unfolded Protein Response: Roles in Skeletal Muscle Atrophy.” Biochemical Pharmacology 234: 116799. 10.1016/j.bcp.2025.116799 39952329

[acel70773-bib-0029] Jia, J. , L. Wang , Y. Zhou , P. Zhang , and X. Chen . 2024. “Muscle‐Derived Extracellular Vesicles Mediate Crosstalk Between Skeletal Muscle and Other Organs.” Frontiers in Physiology 15: 1501957. 10.3389/fphys.2024.1501957 PMC11750798 39844898

[acel70773-bib-0030] Jiang, H. , Y. Ji , T. Shang , et al. 2026. “The cGAS‐STING Signaling Pathway: A Central Regulator and Novel Therapeutic Target in Skeletal Muscle Pathophysiology.” Biochemical Pharmacology 248: 117854. 10.1016/j.bcp.2026.117854 41765111

[acel70773-bib-0031] Jiang, W. , K. Chua , H. K. Tan , E. K. Tan , K. Y. Lin , and H. W. Tang . 2026. “Aging of the Neural Stem Cell Niche: Integration of Local and Muscle‐Derived Systemic Signals in Preclinical and Translational Contexts.” Npj Aging. 10.1038/s41514-026-00468-5.

[acel70773-bib-0032] Kargl, C. K. , B. P. Sullivan , D. Middleton , et al. 2023. “Peroxisome Proliferator‐Activated Receptor Gamma Coactivator 1‐Alpha Overexpression Improves Angiogenic Signalling Potential of Skeletal Muscle‐Derived Extracellular Vesicles.” Experimental Physiology 108: 240–252. 10.1113/EP090874 PMC9949767 36454193

[acel70773-bib-0033] Lee, W. X. , K. Y. Goh , S. M. Choy , and H.‐W. Tang . 2025. “DEAF1 ‐ a Transcriptional Brake on Muscle Autophagy.” Autophagy 22: 640–641. 10.1080/15548627.2025.2610451. PMC13048571 41456123

[acel70773-bib-0034] Li, M. , R. Soder , S. Abhyankar , et al. 2025. “Large‐Scale Manufacturing of Immunosuppressive Extracellular Vesicles for Human Clinical Trials.” Cytotherapy 27: 1219–1228. 10.1016/j.jcyt.2025.06.003 PMC13267737 40586746

[acel70773-bib-0035] Lin, C. C. , J. Rose , A. A. Mestre , et al. 2026. “MESH1‐Mediated Coenzyme A Degradation Drives Ferroptosis Sensitivity and Muscle Pathology.” Journal of Clinical Investigation 136: e202212. 10.1172/JCI202212 PMC13221220 42033660

[acel70773-bib-0036] Lin, J. , X. Shao , T. Shi , et al. 2026. “Exercise Alleviates Cognitive Dysfunction in Alzheimer's Disease Mice via Skeletal Muscle‐Derived Extracellular Vesicles That Enhance Plaque Clearance by Microglia.” Nature Aging 6: 579–596. 10.1038/s43587-026-01075-5 41772068

[acel70773-bib-0037] Liu, H. , X. Zhu , J. Wang , et al. 2026. “Mechanisms and Therapeutic Potential of the cGAS‐STING Signaling Pathway in Macrophage Activation and Polarization.” Biochemical Pharmacology 250: 118031. 10.1016/j.bcp.2026.118031 42082120

[acel70773-bib-0038] Liu, R. , Y. Shen , Y. Wei , et al. 2026. “The Central Role of Macrophages in Skeletal Muscle Regeneration: Phenotypic Polarization, Metabolic Reprogramming, and Therapeutic Prospects.” Biochemical Pharmacology 250: 118025. 10.1016/j.bcp.2026.118025 42069229

[acel70773-bib-0039] Liu, X. , Z. Yao , L. Zhang , and N. Shyh‐Chang . 2025. “Muscle‐Derived Bioactive Factors: MyoEVs and Myokines.” Cell Proliferation 58: e13801. 10.1111/cpr.13801 PMC11882754 39737773

[acel70773-bib-0040] Ma, S. , X. Xing , H. Huang , et al. 2023. “Skeletal Muscle‐Derived Extracellular Vesicles Transport Glycolytic Enzymes to Mediate Muscle‐To‐Bone Crosstalk.” Cell Metabolism 35: 2028–2043.e2027. 10.1016/j.cmet.2023.10.013 37939660

[acel70773-bib-0041] Madison, R. D. , C. McGee , R. Rawson , and G. A. Robinson . 2014. “Extracellular Vesicles From a Muscle Cell Line (C2C12) Enhance Cell Survival and Neurite Outgrowth of a Motor Neuron Cell Line (NSC‐34).” Journal of Extracellular Vesicles 3: 22865. 10.3402/jev.v3.22865 PMC3930942 24563732

[acel70773-bib-0042] Mannaperuma, D. , D. Stratton , S. Lange , and J. M. Inal . 2025. “Extracellular Vesicles From the Myocyte Secretome Contribute In Vitro to Creating an Unfavourable Environment for Migrating Lung Carcinoma Cells.” Biology‐Basel 14: 1578. 10.3390/biology14111578 PMC12650371 41300368

[acel70773-bib-0043] Matthews, I. , A. Birnbaum , A. Gromova , et al. 2023. “Skeletal Muscle TFEB Signaling Promotes Central Nervous System Function and Reduces Neuroinflammation During Aging and Neurodegenerative Disease.” Cell Reports 42: 113436. 10.1016/j.celrep.2023.113436 PMC10841857 37952157

[acel70773-bib-0044] McNally, B. D. , D. F. Ashley , L. Hänschke , et al. 2022. “Long‐Chain Ceramides Are Cell Non‐Autonomous Signals Linking Lipotoxicity to Endoplasmic Reticulum Stress in Skeletal Muscle.” Nature Communications 13: 1748. 10.1038/s41467-022-29363-9 PMC8975934 35365625

[acel70773-bib-0045] Murach, K. A. , I. J. Vechetti , D. W. van Pelt , et al. 2020. “Fusion‐Independent Satellite Cell Communication to Muscle Fibers During Load‐Induced Hypertrophy.” Function (Oxford, England) 1: zqaa009. 10.1093/function/zqaa009 PMC7448100 32864621

[acel70773-bib-0046] Pedersen, B. K. 2019. “Physical Activity and Muscle‐Brain Crosstalk.” Nature Reviews Endocrinology 15: 383–392. 10.1038/s41574-019-0174-x 30837717

[acel70773-bib-0047] Pinto, A. C. , P. Tavares , B. Neves , et al. 2024. “Exploiting the Therapeutic Potential of Contracting Skeletal Muscle‐Released Extracellular Vesicles in Cancer: Current Insights and Future Directions.” Journal of Molecular Medicine (Berlin) 102: 617–628. 10.1007/s00109-024-02427-7 PMC11055777 38451309

[acel70773-bib-0048] Rome, S. , A. Forterre , M. L. Mizgier , and K. Bouzakri . 2019. “Skeletal Muscle‐Released Extracellular Vesicles: State of the Art.” Frontiers in Physiology 10: 929. 10.3389/fphys.2019.00929 PMC6695556 31447684

[acel70773-bib-0049] Severinsen, M. C. K. , and B. K. Pedersen . 2020. “Muscle‐Organ Crosstalk: The Emerging Roles of Myokines.” Endocrine Reviews 41: 594–609. 10.1210/endrev/bnaa016 PMC7288608 32393961

[acel70773-bib-0050] Shang, T. , H. Xu , X. Yao , et al. 2026. “Dual Role of Macrophages in Skeletal Muscle Atrophy: Mechanisms and Therapeutic Strategies.” Biochemical Pharmacology 244: 117589. 10.1016/j.bcp.2025.117589 41344510

[acel70773-bib-0051] Shao, X. , P. Zhang , Z. Fan , et al. 2026. “Atrophic Skeletal Muscle‐Derived Extracellular Vesicles Transfer miR‐125a‐5p to Inhibit Bone Formation in Osteoporosis During Aging.” Advanced Science (Weinheim, Baden‐Wurttemberg, Germany) 13: e15362. 10.1002/advs.202515362. PMC13137847 41747076

[acel70773-bib-0052] Takov, K. , D. M. Yellon , and S. M. Davidson . 2019. “Comparison of Small Extracellular Vesicles Isolated From Plasma by Ultracentrifugation or Size‐Exclusion Chromatography: Yield, Purity and Functional Potential.” Journal of Extracellular Vesicles 8: 1560809. 10.1080/20013078.2018.1560809 PMC6327926 30651940

[acel70773-bib-0053] Vechetti, I. J., Jr. , B. D. Peck , Y. Wen , et al. 2021. “Mechanical Overload‐Induced Muscle‐Derived Extracellular Vesicles Promote Adipose Tissue Lipolysis.” FASEB Journal 35: e21644. 10.1096/fj.202100242R PMC8607211 34033143

[acel70773-bib-0054] Vechetti, I. J., Jr. , T. Valentino , C. B. Mobley , and J. J. McCarthy . 2021. “The Role of Extracellular Vesicles in Skeletal Muscle and Systematic Adaptation to Exercise.” Journal of Physiology 599: 845–861. 10.1113/JP278929 PMC7363579 31944292

[acel70773-bib-0055] Vints, W. A. J. , O. Levin , H. Fujiyama , J. Verbunt , and N. Masiulis . 2022. “Exerkines and Long‐Term Synaptic Potentiation: Mechanisms of Exercise‐Induced Neuroplasticity.” Frontiers in Neuroendocrinology 66: 100993. 10.1016/j.yfrne.2022.100993 35283168

[acel70773-bib-0056] Vitucci, D. , D. Martone , A. Alfieri , and P. Buono . 2023. “Muscle‐Derived Exosomes and Exercise in Cancer Prevention.” Frontiers in Molecular Medicine 3: 1202190. 10.3389/fmmed.2023.1202190 PMC11285545 39086668

[acel70773-bib-0057] Wang, J. , J. Gu , X. Li , H. Sun , and X. Yang . 2026. “The Central Role of the AMPK/SIRT1/PGC‐1alpha Signaling Axis in Skeletal Muscle Physiology and Pathology and Its Targeted Therapeutic Strategies.” Pharmaceuticals 19: 1056. 10.3390/ph19071056 PMC13414853 42515737

[acel70773-bib-0058] Welsh, J. A. , D. C. I. Goberdhan , L. O'Driscoll , et al. 2024. “Minimal Information for Studies of Extracellular Vesicles (MISEV2023): From Basic to Advanced Approaches.” Journal of Extracellular Vesicles 13: e12404. 10.1002/jev2.12404 PMC10850029 38326288

[acel70773-bib-0059] Xhuti, D. , M. I. Nilsson , K. Manta , M. A. Tarnopolsky , and J. P. Nederveen . 2023. “Circulating Exosome‐Like Vesicle and Skeletal Muscle microRNAs Are Altered With Age and Resistance Training.” Journal of Physiology 601: 5051–5073. 10.1113/JP282663 36722691

[acel70773-bib-0060] Xu, Q. , Y. Cui , J. Luan , X. Zhou , H. Li , and J. Han . 2018. “Exosomes From C2C12 Myoblasts Enhance Osteogenic Differentiation of MC3T3‐E1 Pre‐Osteoblasts by Delivering miR‐27a‐3p.” Biochemical and Biophysical Research Communications 498: 32–37. 10.1016/j.bbrc.2018.02.144 29476741

[acel70773-bib-0061] Xue, F. , Y. Ji , J. Wang , X. Li , B. Dai , and H. Sun . 2026. “The Role of Piezo1 in Skeletal Muscle Physiology and Pathology: From Mechanosensing to Precision Therapy.” Biochemical Pharmacology 252: 118219. 10.1016/j.bcp.2026.118219 42402277

[acel70773-bib-0062] Yamaguchi, A. , N. Maeshige , J. Yan , et al. 2023. “Skeletal Myotube‐Derived Extracellular Vesicles Enhance Itaconate Production and Attenuate Inflammatory Responses of Macrophages.” Frontiers in Immunology 14: 1099799. 10.3389/fimmu.2023.1099799 PMC10018131 36936950

[acel70773-bib-0063] Yan, Y. , M. Li , J. Lin , et al. 2022. “Adenosine Monophosphate Activated Protein Kinase Contributes to Skeletal Muscle Health Through the Control of Mitochondrial Function.” Frontiers in Pharmacology 13: 947387. 10.3389/fphar.2022.947387 PMC9632297 36339617

[acel70773-bib-0064] Yang, C. P. , W. S. Yang , Y. H. Wong , et al. 2020. “Muscle Atrophy‐Related Myotube‐Derived Exosomal microRNA in Neuronal Dysfunction: Targeting Both Coding and Long Noncoding RNAs.” Aging Cell 19: e13107. 10.1111/acel.13107 PMC7253071 32233025

[acel70773-bib-0065] Zhang, L. , M. Li , W. Wang , et al. 2022. “Celecoxib Alleviates Denervation‐Induced Muscle Atrophy by Suppressing Inflammation and Oxidative Stress and Improving Microcirculation.” Biochemical Pharmacology 203: 115186. 10.1016/j.bcp.2022.115186 35882305

[acel70773-bib-0066] Zhou, J. , P. Chen , X. Chen , et al. 2026. “Label‐Free Clustering Analysis Platform Drives Cascaded Workflow for Scalable Production of Therapeutic Extracellular Vesicles.” Journal of Extracellular Vesicles 15: e70333. 10.1002/jev2.70333 PMC13291197 42338032

